# Predictors of Early Mortality After Open Aorto-Iliac Reconstruction in Patients with Aortoiliac Occlusive Disease: An Observational Cohort Study

**DOI:** 10.3390/medicina62102001

**Published:** 2026-10-16

**Authors:** Merei Imanbayev, Altay Dyussupov, Bekzhigit Tussupkanov, Ergali Muratzhanov, Ardak Mautbekova, Dinara Kozubayeva, Rustam Mukhametkhanov, Rail Gafurov, Nurbek Irgebayev, Dainius Pavalkis, Evgeniy Zhuravlev, Yersyn Sabitov

**Affiliations:** 1 Department of Cardiovascular and Thoracic Surgery Named After B.S. Bulanov, NCJSC Semey Medical University, 103 Abai Street, Semey 071400, Kazakhstan; altay.dyusupov@smu.edu.kz (A.D.); tusupkanov260398@gmail.com (B.T.); argyn.nurbek@mail.ru (N.I.); 2 University Hospital Semey, NCJSC Semey Medical University, Semey 071400, Kazakhstan; m.e.t98@mail.ru (E.M.); mautbekova.ardak@mail.ru (A.M.); mrbkz@mail.ru (R.M.); saderikaa@mail.ru (R.G.); anesteziolog2008@mail.ru (E.Z.); 3 Department of Infectious Diseases, Dermatovenerology and Immunology, NCJSC Semey Medical University, 103 Abai Street, Semey 071400, Kazakhstan; dinara_begimhanovna@mail.ru; 4 Research and Innovation Department, Lithuanian University of Health Sciences, A.Mickeviciaus Str. 9, 44307 Kaunas, Lithuania; Dainius.Pavalkis@lsmu.lt; 5 Municipal State Enterprise on the Right of Economic Management “Emergency Medical Hospital of Semey”, Semey 071404, Kazakhstan

**Keywords:** aorto-iliac occlusive disease, open aorto-iliac reconstruction, 30-day mortality, late mortality, internal iliac artery stenosis, chronic kidney disease, aortic cross-clamp time, Cox proportional hazards regression

## Abstract

*Background and Objectives*: Open aorto-iliac reconstruction (AIR) for aorto-iliac occlusive disease (AIOD) remains a high-risk procedure due to multifocal atherosclerosis affecting coronary, cerebrovascular, and renal arterial beds. This study aimed to identify factors independently associated with 30-day mortality and explore factors associated with late mortality during available follow-up among 30-day survivors following open AIR. *Materials and Methods*: An observational, non-randomised cohort of 170 consecutive patients undergoing open AIR for AIOD between January 2018 and December 2024 was analysed. Univariate logistic regression was performed for 30-day mortality; given the limited number of events (*n* = 16), the primary multivariable analysis used penalized likelihood logistic regression (Firth correction). Late survival among 30-day survivors (landmark cohort, *n* = 154) was analysed using Kaplan–Meier methods and exploratory univariate Cox proportional hazards regression, with surviving patients censored at the last available follow-up; multivariable Cox modelling was not performed due to the limited number of late events (*n* = 10). *Results*: Thirty-day mortality was 9.4% (16/170) in the overall cohort. In the primary Firth penalized multivariable logistic regression, factors associated with 30-day mortality were: severe left IIA stenosis ≥ 70% (OR 9.68; 95% CI 3.08–30.43; *p* = 0.0001), chronic kidney disease (OR 3.62; 95% CI 1.03–12.66; *p* = 0.044), and increasing aortic cross-clamp duration, analysed continuously (OR 1.022 per minute; 95% CI 1.000–1.045); a conventional ≥60 min categorical threshold showed a consistent secondary association (OR 4.15; 95% CI 1.28–13.46; *p* = 0.018). Non-inclusion of IIA was associated with increased 30-day mortality on univariate analysis (OR 4.80; 95% CI 1.31–17.62; *p* = 0.018). Among the 154 patients entering the 30-day landmark cohort, 10 late deaths were observed during available follow-up. In exploratory univariate Cox regression, intraoperative blood loss was the only statistically significant factor associated with late mortality (HR 1.07 per 100 mL; 95% CI 1.03–1.12; *p* = 0.0006). *Conclusions*: Severe IIA disease, chronic kidney disease, and increasing aortic cross-clamp duration (analysed continuously) were independently associated with increased 30-day mortality after open AIR. Intraoperative blood loss was associated with late mortality in exploratory univariate survival analysis. These findings require validation in larger prospective cohorts with longer and more uniform follow-up.

## 1. Introduction

Aorto-iliac occlusive disease (AIOD) represents one of the most prevalent manifestations of peripheral arterial disease, affecting 0.9–7% of the population depending on age and risk factor burden [1,2]. The pathological substrate is atherosclerosis, which characteristically presents with multifocal involvement of the arterial tree, exposing affected patients concurrently to coronary artery disease (CAD), cerebrovascular disease, and renal dysfunction [3,4].

Open aorto-iliac reconstruction (AIR)–encompassing bifurcation aorto-femoral bypass, linear aorto-femoral bypass, and aorto-ilio-femoral reconstruction–has long been the gold standard for definitive revascularisation of TASC II C/D lesions not amenable to endovascular repair [5]. Despite decades of refinement, open AIR carries a 30-day mortality of 2–8% and 5-year survival of 60–80%, driven primarily by the burden of concomitant atherosclerosis of vital organ arterial beds [6,7,8].

A substantial proportion of AIOD patients are anatomically suitable for endovascular stenting [4,9]. The remainder require open surgery, often under time pressure due to critical limb ischaemia with imminent irreversible trophic change. Accurate identification of factors associated with early and late mortality, particularly potentially modifiable perioperative factors, is essential for surgical planning and patient counselling.

The present study aimed to: (1) characterise 30-day mortality and late mortality during available follow-up among 30-day survivors in an observational cohort of open AIR patients; (2) identify factors independently associated with early mortality and explore factors associated with late mortality; and (3) assess the contribution of internal iliac artery (IIA) inclusion category to perioperative mortality.

## 2. Materials and Methods

### 2.1. Study Design and Setting

An observational, non-randomised cohort study was conducted at the Department of Cardiovascular Surgery, University Hospital of NCJSC Semey Medical University, Semey, Republic of Kazakhstan. The study included consecutive eligible patients who underwent open aorto-iliac reconstruction (AIR) for aortoiliac occlusive disease between January 2018 and December 2024. Clinical, imaging, operative, and postoperative data were obtained from the institutional clinical database and medical records. Outcome ascertainment was based on scheduled clinical follow-up and institutional medical records.

**Figure 1:**
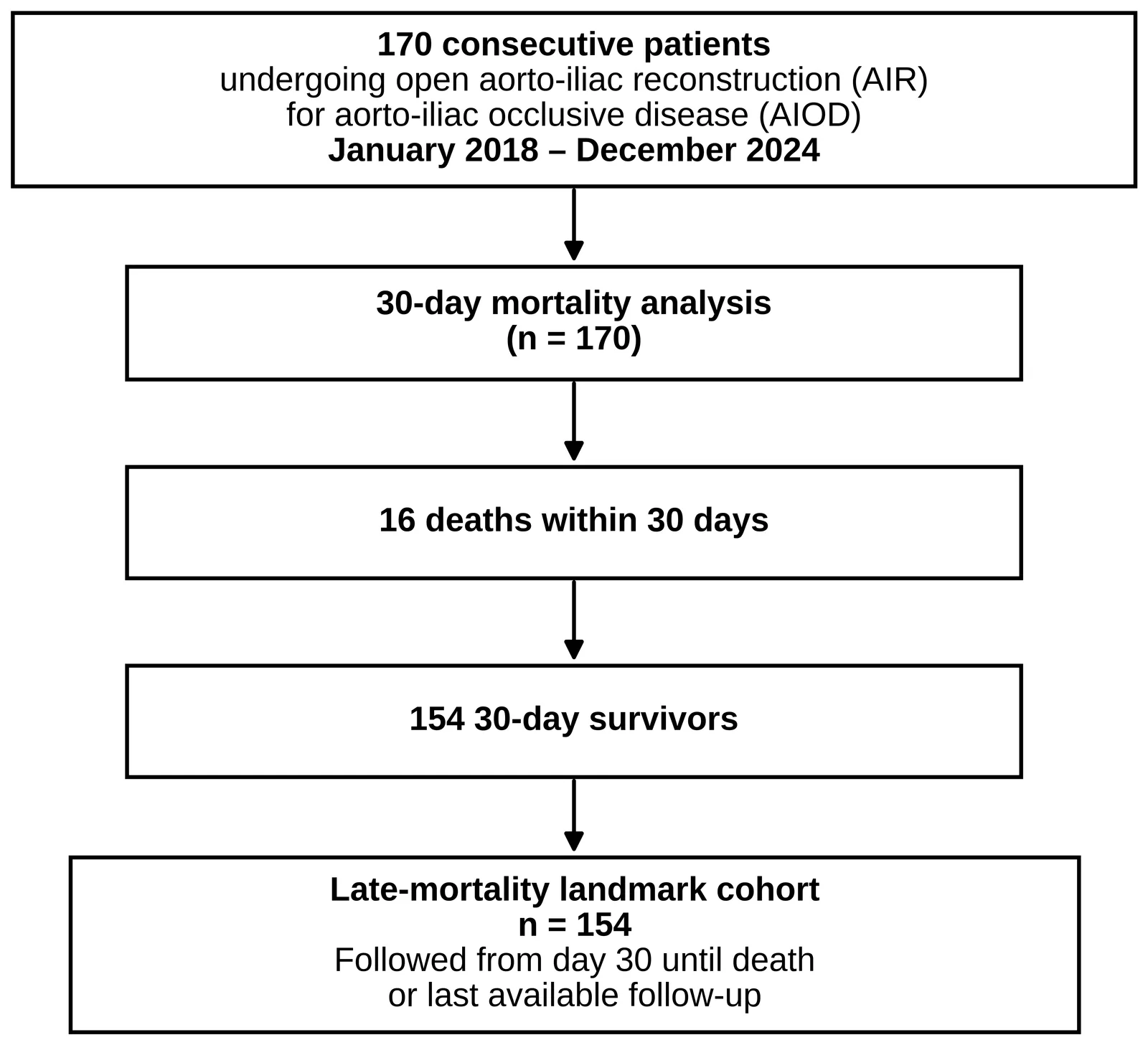
Study flow diagram. A total of 170 consecutive patients undergoing open aorto-iliac reconstruction for aortoiliac occlusive disease between January 2018 and December 2024 were included in the 30-day mortality analysis. Sixteen patients died within 30 days after surgery and were excluded from the late-mortality landmark analysis. The remaining 154 30-day survivors entered the landmark cohort and were followed from day 30 until death or the last available follow-up.

The study flow diagram is presented in Figure 1. Of the 170 patients included in the present study, 134 had been included in a previous publication from our institution [10]. The present study included 36 additional patients and addressed a distinct research objective focused on predictors of mortality using a substantially different statistical approach.

The study was approved by the Institutional Ethics Committee of Semey Medical University (Protocol No. 4, dated 27 September 2017) and was conducted in accordance with the Declaration of Helsinki. All participants provided written informed consent for participation in the study.

### 2.2. Participants

Inclusion criteria: confirmed occlusive lesion of the aorto-iliac segment; planned or urgent open AIR; open reconstruction performed. Exclusion criteria: juxta- or inter-renal aortic occlusion; abdominal aortic aneurysm; emergency surgery for aortic rupture; endovascular aorto-iliac replacement.

Treatment strategy was recorded as a clinical characteristic. Of the 170 patients, 86 underwent urgent AIR without staged preoperative correction of comorbidities, whereas 84 underwent AIR after staged preoperative correction of haemodynamically significant coronary artery disease (CABG or PTCA), carotid stenosis (CEA), and/or renal dysfunction. Because treatment allocation was non-randomised and the two treatment-strategy subgroups differed substantially in baseline characteristics (Supplementary Table S2), treatment strategy was not considered the primary exposure or basis for comparative inference and was retained only as a covariate in the mortality analyses.

### 2.3. Preoperative Assessment

Preoperative workup included: clinical examination; laboratory tests (complete blood count, serum creatinine, coagulation profile); ECG; echocardiography with LVEF measurement; Doppler ultrasound with duplex scanning; multi-slice computed tomography angiography (MSCTA); coronary angiography; and transcranial Doppler sonography when indicated.

### 2.4. Surgical Techniques

AIR was performed via standard midline transperitoneal approach under general anaesthesia using Dacron prostheses. Techniques: bifurcation aorto-femoral bypass; linear aorto-femoral bypass; bifurcation aorto-ilio-femoral bypass; and bifurcation aorto-femoral bypass with IIA revascularisation. IIA inclusion was categorized at the patient level into three mutually exclusive groups according to the type of aorto-iliac reconstruction recorded in the operative dataset. Antegrade IIA inclusion comprised linear aorto-femoral bypass (*n* = 37), bifurcation aorto-ilio-femoral bypass (*n* = 10), and bifurcation aorto-femoral bypass with IIA revascularisation (*n* = 39). Retrograde IIA inclusion comprised bifurcation aorto-femoral bypass without IIA revascularisation (*n* = 70). Non-inclusion was recorded in 14 patients (bifurcation aorto-femoral bypass, *n* = 13; bifurcation aorto-femoral bypass with IIA revascularisation, *n* = 1). The configuration of the proximal anastomosis (end-to-side versus end-to-end) and the specific technique used for IIA revascularisation were not recorded as separate variables in the historical dataset. Reimplantation of the inferior mesenteric artery was recorded separately and was independent of the IIA inclusion classification.

### 2.5. Outcome Definitions

The primary outcome was 30-day all-cause mortality, defined as death from any cause within 30 days after the index AIR procedure. The secondary outcome was late all-cause mortality among patients who survived beyond 30 days. For the late-mortality analysis, day 30 after surgery was used as the landmark. Follow-up duration varied according to the date of enrolment. Patients were followed from the 30-day landmark until death, the last available clinical contact, or the end of the observation period, whichever occurred first. Patients without a recorded death were censored at their last known follow-up. Because enrolment continued through December 2024, not all patients had the opportunity to complete 5 years of follow-up.

### 2.6. Statistical Analysis

Statistical analyses were performed using IBM SPSS Statistics (version 20.0; IBM Corp., Armonk, NY, USA), with additional and sensitivity analyses conducted in Python (version 3.12) using the SciPy, statsmodels, and lifelines libraries. Normally distributed continuous data are presented as mean ± SD; non-normal data as median [IQR]. Normality was assessed by Shapiro–Wilk test (*n* ≤ 50) or Kolmogorov–Smirnov test and Q–Q plots. Between-group comparisons used Student’s *t*-test or Mann–Whitney U test (continuous) and chi-squared or Fisher’s exact test (categorical).

For the 30-day mortality endpoint, univariate logistic regression was first performed. Given the limited number of early deaths (*n*  =  16) and the risk of small-sample bias and model overfitting, the primary multivariable analysis was performed using penalized likelihood logistic regression (Firth correction). The final model included clinically motivated predictor domains—internal iliac artery (IIA) stenosis severity, chronic kidney disease, and aortic cross-clamp duration—with smoking added as a fourth covariate because of its borderline univariate association with 30-day mortality (OR 7.50; *p*  =  0.054). These predictor domains were clinically motivated rather than fully prespecified, and their specific operationalisation was refined using the observed data rather than being fixed a priori: left IIA stenosis was selected over bilateral or right IIA stenosis because it demonstrated the strongest association with 30-day mortality in preliminary univariate analyses (AUC 0.763 vs. 0.733 right IIA; AUC 0.765 mean bilateral) and because of collinearity between unilateral and bilateral stenosis measures, and the categorical thresholds described below were likewise chosen using a combination of established clinical criteria and the observed data. This data-informed model selection is acknowledged as a limitation of the analysis in Section 4.6.

The ≥70% IIA stenosis threshold was used as a clinically interpretable definition of severe IIA stenosis, informed by studies of IIA flow and pelvic perfusion in aorto-iliac reconstruction [11]; it was not derived from a formally validated diagnostic threshold specific to AIOD. The discriminatory performance of left IIA stenosis was additionally explored using ROC analysis (AUC 0.763; optimal cut-off 65%; sensitivity 0.750, specificity 0.817). Because ROC analysis identified 65% as the data-derived optimum rather than 70%, the ≥70% categorical definition should be understood as a pragmatic clinical convention rather than an externally validated cut-off; a continuous-variable sensitivity analysis is described below.

Although ROC analysis for cross-clamp time identified 50 min as the mathematical optimum (AUC 0.651; sensitivity 75.0%; specificity 52.6%), a ≥60 min threshold was additionally examined for clinical interpretability as a conventional, easily communicated round-number cut-off; we did not identify a specific published threshold to validate this choice externally, and it should therefore be regarded as a pragmatic rather than an evidence-based cut-off. Given the absence of an externally validated threshold, aortic cross-clamp duration was analysed continuously as the primary representation of this predictor; the ≥60 min threshold was retained only as a secondary categorical analysis for clinical interpretability. Continuous-variable sensitivity analyses were also performed for the other predictors above. For the analysis of late mortality, a landmark approach was used. Patients who died within the first 30 postoperative days were excluded, leaving 154 patients in the landmark cohort. Time zero was defined as day 30 after the index procedure. Patients were followed from the landmark until death or censoring at the last available follow-up. Kaplan–Meier methods were used to describe late survival, and exploratory univariate Cox proportional hazards regression was used to assess associations with late mortality. Because only 10 late deaths were observed, multivariable Cox regression was not performed to avoid model instability and overfitting. One patient (0.6%) had a missing value for left internal iliac artery stenosis and was excluded from the multivariable Firth logistic regression model (complete-case analysis, *n* = 169); no other missing data were present among the variables included in the primary analyses. No formal a priori sample size calculation was performed; all consecutive eligible patients treated during the study period were included. Statistical significance was defined as *p* < 0.05 (two-tailed). This study was reported in accordance with the STROBE guidelines for observational cohort studies.

## 3. Results

### 3.1. Baseline Characteristics

The cohort (*n* = 170) was predominantly male (157/170, 92.4%), with a mean age of 63.8 ± 9.4 years. Smoking was prevalent (117/170, 68.8%). Atherosclerosis was the aetiological cause of AIOD in all cases. Full baseline characteristics for the overall cohort are shown in Table 1. Patients also differed by treatment strategy (Section 2.2); because of substantial baseline imbalance between these subgroups—particularly in the prevalence of ischaemic heart disease, prior myocardial infarction (PICS), arterial hypertension, and CKD—treatment-strategy comparisons are presented descriptively in Supplementary Table S2 rather than as a primary between-group analysis (see Limitations, Section 4.6).

### 3.2. Operative Details

Mean aortic cross-clamp time was 51.6 ± 22.4 min (range 21–160 min). Mean intraoperative blood loss was 647 ± 564 mL. IIA inclusion category was antegrade in 86/170 (50.6%), retrograde in 70/170 (41.2%), and non-inclusion in 14/170 (8.2%). Reconstruction type and IIA inclusion category are presented for the overall cohort in Table 2. IIA inclusion category is included as a covariate in the mortality analyses; it also differed by treatment strategy, reflecting differences in anatomy, urgency, and surgical selection between the urgent- and staged-surgery subgroups (Supplementary Table S3).

### 3.3. Early (30-Day) Mortality

Sixteen patients (9.4%) died within 30 days. Causes of death: acute myocardial infarction (*n* = 4), acute renal failure (*n* = 4), stroke (*n* = 3), multiple organ failure (*n* = 3), and other (*n* = 2).

#### 3.3.1. Univariate Logistic Regression

Factors associated with 30-day mortality in univariate logistic regression included non-inclusion of the IIA (OR 4.80; *p* = 0.018), right IIA stenosis (OR 1.025 per 1%; *p* = 0.001), and left IIA stenosis (OR 1.029 per 1%; *p* = 0.0002); smoking showed a non-significant trend (OR 7.50; *p* = 0.054). All results are shown in Table 3.

**Table 3:** Univariate logistic regression analysis of factors associated with 30-day mortality.

Variable	OR	95% CI	*p*
Non-inclusion of IIA	4.80	1.31–17.62	0.018
IIA stenosis right (per 1%)	1.025	1.010–1.040	0.001
IIA stenosis left (per 1%)	1.029	1.014–1.045	0.0002
Aortic cross-clamp time (per min)	1.014	0.995–1.033	0.162
Chronic kidney disease	2.24	0.72–6.98	0.165
Smoking	7.50	0.96–58.37	0.054
Antegrade IIA inclusion	0.41	0.14–1.24	0.113
Age (per year)	1.004	0.949–1.063	0.877
LVEF (per %)	0.981	0.924–1.042	0.536
Prior MI (PICS)	1.55	0.53–4.52	0.424
Treatment strategy (staged vs. urgent surgery)	0.86	0.26–2.82	0.804

IIA: internal iliac artery; LVEF: left ventricular ejection fraction; MI: myocardial infarction; PICS: postinfarction cardiosclerosis; OR: odds ratio; CI: confidence interval.

#### 3.3.2. Multivariable Logistic Regression

Severe left IIA stenosis (≥70%) was present in 36 of 170 patients (21.2%), of whom 10 died within 30 days (30-day mortality 27.8% vs. 4.5% in patients with left IIA stenosis <70%; *p* < 0.001). In the primary Firth penalized multivariable logistic regression model, severe left IIA stenosis ≥ 70% remained strongly associated with 30-day mortality (OR 9.68; 95% CI 3.08–30.43; *p* = 0.0001). Chronic kidney disease was also independently associated with increased early mortality (OR 3.62; 95% CI 1.03–12.66; *p* = 0.044). Increasing aortic cross-clamp duration, analysed continuously as the primary representation of this predictor, was associated with a higher risk of 30-day mortality (OR 1.022 per minute; 95% CI 1.000–1.045). A conventional ≥60 min categorical threshold, retained as a secondary, clinically interpretable analysis, showed a consistent association (OR 4.15; 95% CI 1.28–13.46; *p* = 0.018). These findings were also consistent with the continuous-variable sensitivity estimates for the other predictors (left IIA stenosis per 1%: OR 1.031, 95% CI 1.015–1.047; CKD: OR 4.58, 95% CI 1.23–17.04). Results are shown in Table 4. ROC analysis for model discrimination showed adequate predictive ability for left IIA stenosis (AUC = 0.763; optimal cut-off 65%; sensitivity 75.0%; specificity 81.7%) and right IIA stenosis (AUC = 0.733). Aortic cross-clamp time had an AUC of 0.651 (optimal cut-off 50 min; sensitivity 75.0%).

**Table 4:** Firth penalized multivariable logistic regression analysis of factors associated with 30-day mortality (*n* = 169; one patient excluded due to a missing left IIA stenosis value).

Variable	OR	95% CI	*p*
Left IIA stenosis ≥ 70%	9.68	3.08–30.43	0.0001
Chronic kidney disease	3.62	1.03–12.66	0.044
Aortic cross-clamp time ≥ 60 min	4.15	1.28–13.46	0.018
Smoking	4.07	0.48–34.32	0.197

IIA: internal iliac artery; CKD: chronic kidney disease; OR: odds ratio; CI: confidence interval. Note: aortic cross-clamp duration was primarily interpreted as a continuous predictor; the ≥60 min categorisation was retained as a secondary, clinically interpretable analysis. Continuous-variable sensitivity analyses were also performed for the other predictors.

### 3.4. Late Mortality Among 30-Day Survivors

**Figure 2:**
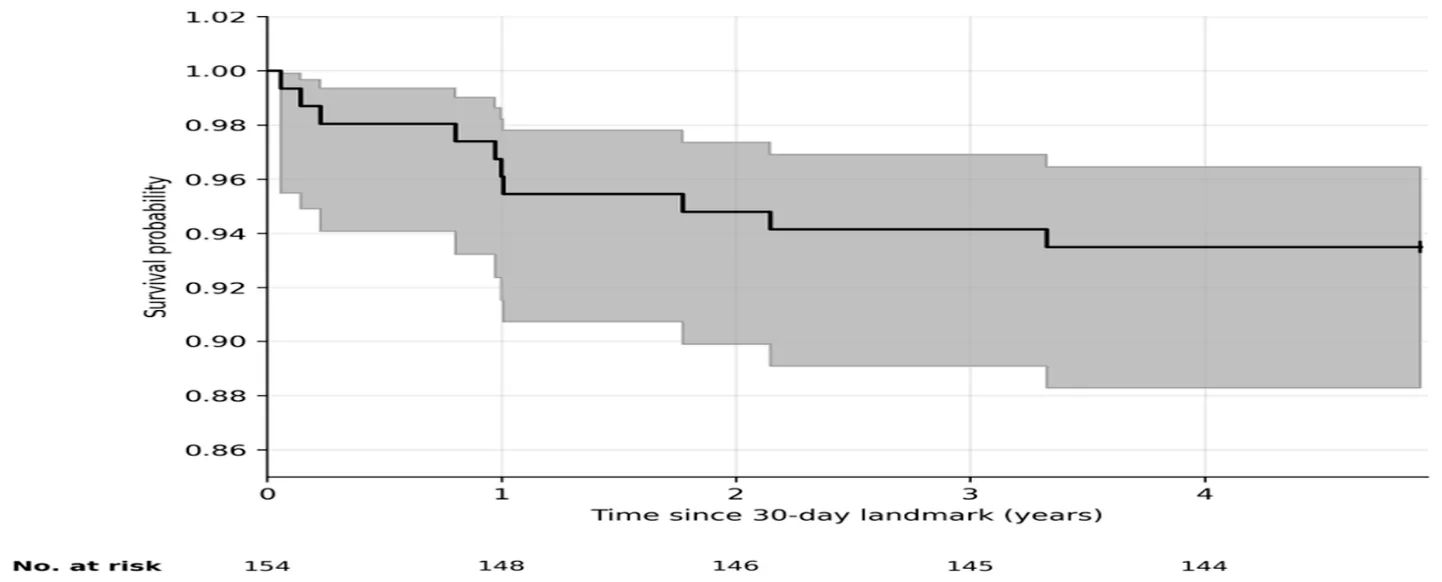
Censored Kaplan–Meier curve for late survival among the 30-day landmark cohort (*n* = 154; 10 late deaths), with time zero defined as day 30 after the index procedure. Shaded area shows the 95% confidence band; vertical tick marks indicate censored observations. The number of patients at risk at each landmark year is shown below the plot. Maximum available landmark follow-up was 4.9 years (censoring at 5 years post-surgery, i.e., 30 days after the landmark start); no patient reached a full 5 years of landmark follow-up, consistent with the heterogeneous enrolment period (January 2018–December 2024) described in the Limitations section.

Among the 154 patients who survived beyond 30 days and entered the landmark analysis, 10 late deaths were observed during available follow-up. Causes of late death: MI (*n* = 3), renal failure (*n* = 3), stroke (*n* = 2), oncological pathology (*n* = 1), sudden death (*n* = 1). On univariate Cox regression, intraoperative blood loss was the only statistically significant factor associated with late mortality during available follow-up in this exploratory analysis (HR 1.07 per 100 mL; 95% CI 1.03–1.12; *p* = 0.0006). Because of the small number of late deaths (*n*  =  10), multivariable Cox regression was not performed, as the model would be statistically unstable and prone to overfitting (Table 5). The corresponding censored Kaplan–Meier survival curve for this landmark cohort, together with the number of patients at risk at each year of follow-up, is shown in Figure 2.

**Table 5:** Univariate Cox proportional hazards regression analysis of factors associated with late mortality among 30-day survivors (landmark cohort, *n* = 154).

Variable	HR	95% CI	*p*
Blood loss (per 100 mL)	1.07	1.03–1.12	0.0006
Smoking	0.48	0.14–1.66	0.246
IIA stenosis left (per 1%)	0.985	0.960–1.012	0.278
Chronic kidney disease	2.13	0.55–8.22	0.275
Treatment strategy (staged vs. urgent surgery)	0.99	0.29–3.42	0.988
Prior MI (PICS)	1.77	0.50–6.26	0.379
Age (per year)	0.992	0.932–1.057	0.814
LVEF (per %)	0.980	0.914–1.050	0.567

HR: hazard ratio; CI: confidence interval; MI: myocardial infarction; PICS: postinfarction cardiosclerosis; LVEF: left ventricular ejection fraction. Time zero was defined as day 30 after the index procedure; follow-up continued until death, with surviving patients censored at the last available follow-up or at the end of the observation period, as applicable. Analysis restricted to the landmark cohort of 154 patients who survived beyond 30 days (10 late-mortality events); multivariable Cox model not reported due to insufficient events. Postoperative myocardial infarction and postoperative renal complications were excluded from this table because they are post-baseline, time-varying events rather than fixed baseline covariates; including them as ordinary predictors in a standard Cox model would risk immortal-time bias and would require a time-dependent covariate analysis, which the available event numbers cannot support.

## 4. Discussion

This observational cohort study of 170 patients undergoing open AIR for AIOD identified three principal findings: (1) severe left IIA stenosis ≥70% was independently associated with 30-day mortality in the multivariable model, whereas IIA non-inclusion was associated with mortality only in univariate analysis, together suggesting a possible role for systematic preoperative IIA quantification and pelvic flow preservation; (2) pre-existing CKD is independently associated with increased 30-day mortality (OR 3.62; Firth corrected); and (3) intraoperative blood loss was the only statistically significant factor associated with late mortality during available follow-up in exploratory univariate Cox regression—a hypothesis-generating finding requiring confirmation in larger cohorts.

### 4.1. IIa and Early Mortality

Although endovascular treatment has become the preferred strategy for many TASC II lesions, open reconstruction remains indispensable in selected patients with extensive aorto-iliac occlusive disease. Mortality following open aortic reconstruction has also been examined in earlier vascular surgical cohorts [12]. The IIA is the principal arterial supply to the sigmoid colon, rectum, urinary bladder, and pelvic musculature. Failure to restore IIA flow during AIR predisposes to ischaemic colitis, pelvic ischaemia, and downstream sepsis or multiple organ failure [10,11,13,14,15]. Non-inclusion of the IIA (OR 4.80 in univariate analysis) and increasing right and left IIA stenosis severity (OR 1.025 and 1.029 per 1% increment, respectively) were associated with 30-day mortality in univariate analyses. Few previous studies have specifically evaluated whether severe IIA disease is independently associated with early mortality, distinct from the intraoperative IIA exclusion strategy. These findings extend previous work from our institution on complication prevention in open aorto-iliac surgery [16] and suggest that systematic preoperative quantification of bilateral IIA stenosis may warrant consideration as part of angiographic workup and surgical planning.

### 4.2. Chronic Kidney Disease

CKD was independently associated with 30-day mortality (OR 3.62; 95% CI 1.03–12.66; *p* = 0.044; Firth penalized model). These findings are consistent with large registry data confirming that CKD severity is an independent predictor of perioperative and 1-year mortality after open aortic repair [17,18]. Patients with AIOD are predisposed to renal dysfunction through renovascular atherosclerosis, contrast nephropathy, and microembolic renal injury during aortic manipulation [19]. The perioperative ‘second hit’ of suprarenal clamping and haemodynamic instability compounds pre-existing renal insufficiency. These findings reinforce the importance of preoperative renal function assessment and support consideration of targeted nephroprotection protocols and staged renal revascularisation where indicated.

### 4.3. Aortic Cross-Clamp Time

Increasing aortic cross-clamp duration was associated with increased 30-day mortality when analysed continuously (OR 1.022 per additional minute; 95% CI 1.000–1.045), which we treat as the primary result for this predictor because it does not depend on an externally unvalidated cut-off; a conventional ≥60 min categorical threshold showed a consistent secondary association (OR 4.15; 95% CI 1.28–13.46; *p* = 0.018). Aortic clamping initiates ischaemia–reperfusion injury characterised by myocardial strain, cytokine release, and acute tubular injury proportional to clamp duration [20]. A mean clamp time of 51.6 ± 22.4 min with a maximum of 160 min indicates substantial variance. These findings suggest that prolonged cross-clamp duration may represent a marker of increased perioperative risk. Whether strategies aimed at reducing cross-clamp time can improve mortality requires prospective evaluation.

### 4.4. Blood Loss and Late Mortality

Intraoperative blood loss was the only statistically significant factor associated with late mortality during available follow-up in exploratory univariate Cox regression (HR 1.07 per 100 mL; 95% CI 1.03–1.12; *p* = 0.0006). This association is unadjusted and derived from only 10 late-mortality events in the landmark cohort, and should therefore be regarded as hypothesis-generating rather than causal; residual confounding (for example, by surgical complexity, intraoperative complications, or comorbidity burden not captured in this model) cannot be excluded. Plausible mechanisms that have been proposed to link intraoperative blood loss to long-term outcomes include coagulopathy, allogeneic transfusion-associated immunosuppression, organ hypoperfusion, and prolonged ICU stay, which may contribute to accelerated atherosclerosis progression and heightened susceptibility to infection [21,22]; however, this small, unadjusted analysis cannot establish which, if any, of these mechanisms apply here. If replicated in larger, adjusted analyses, intraoperative haemostasis optimisation–including cell salvage, antifibrinolytics, and restrictive transfusion strategies–would represent a plausible modifiable target warranting prospective evaluation.

### 4.5. Carotid Stenosis and Ischaemic Heart Disease

Ischaemic heart disease was highly prevalent in the overall cohort (141/170, 82.9%). Myocardial infarction was among the leading causes of both 30-day mortality (4 of 16 early deaths, 25%) and late mortality (3 of 10 late deaths, 30%), highlighting the substantial coronary disease burden among patients undergoing open AIR. These findings support routine, systematic preoperative cardiac risk stratification in AIOD patients regardless of the urgency of presentation. Baseline cardiovascular comorbidity, including the prevalence of ischaemic heart disease and prior myocardial infarction, differed between treatment-strategy subgroups, consistent with non-random treatment allocation and potential confounding by indication (Supplementary Table S2); patients undergoing staged surgery received more extensive preoperative cardiac work-up, including coronary angiography and staged revascularisation, prior to elective AIR (Section 2.2).

Carotid artery stenosis ≥ 50% (either side) was present in 22 of 170 patients (12.9%; Table 1), of whom 17 (77%) had no prior history of stroke and were therefore asymptomatic at the time of the index procedure; the remaining 5 patients had both significant carotid stenosis and a prior stroke. Stroke accounted for 3 of 16 early deaths (18.8%) and 2 of 10 late deaths (20%) in this cohort. Given the very small number of stroke-related deaths, we did not formally test the association between carotid stenosis severity and stroke-related mortality, and this observation should be regarded as descriptive and hypothesis-generating rather than a validated finding. Nonetheless, these findings are consistent with the rationale for the staged preoperative correction strategy applied in the staged-surgery subgroup, in which carotid endarterectomy was performed for haemodynamically significant, predominantly asymptomatic carotid disease prior to elective AIR (Section 2.2).

### 4.6. Limitations

This study has several limitations. First, the non-randomised design and urgency-based treatment allocation introduce substantial confounding by indication. The marked baseline differences between groups, particularly in the prevalence of CKD and prior myocardial infarction (Supplementary Table S2), preclude causal interpretation of between-group comparisons; treatment-group findings should therefore be interpreted descriptively rather than as estimates of the effect of staged preoperative optimisation, and treatment strategy was retained in the mortality analyses only as a covariate (Table 3 and Table 5) rather than as the primary organising comparison of the study, in line with the overall-cohort presentation in Table 1. Second, predictor selection and the operationalisation of some continuous variables were partly data-informed rather than fully prespecified. In particular, left IIA stenosis was selected among correlated IIA measures partly on the basis of its observed association with mortality, while the categorical thresholds for IIA stenosis and aortic cross-clamp duration were pragmatic clinical cut-offs rather than externally validated thresholds. Accordingly, these multivariable findings should be considered exploratory and require external validation. Third, follow-up duration was heterogeneous because enrolment extended through December 2024; consequently, not all participants had the opportunity to complete 5 years of observation. The late-mortality analysis therefore used time-to-event methods with censoring at the last available follow-up rather than assuming uniform follow-up duration. Fourth, detailed GFR staging was not uniformly available across the study period. Fifth, the single-centre design may limit the external generalisability of the findings. Sixth, screening and exclusion counts before cohort entry were not systematically recorded; therefore, a complete pre-enrolment screening flow could not be reconstructed. Seventh, because proximal anastomotic configuration was not recorded separately, the reconstruction-based IIA inclusion categories may not fully represent the physiological direction of IIA perfusion. In particular, preserved native antegrade IIA inflow in the presence of an end-to-side proximal anastomosis cannot be excluded in some patients classified as having retrograde IIA inclusion. This limitation may have resulted in misclassification and should be considered when interpreting associations involving IIA inclusion category.

## 5. Conclusions

In this observational cohort of 170 patients undergoing open aorto-iliac reconstruction for AIOD, severe left IIA stenosis, chronic kidney disease, and increasing aortic cross-clamp duration were independently associated with increased 30-day mortality in multivariable analysis. Non-inclusion of the IIA was associated with increased 30-day mortality on univariate analysis. Intraoperative blood loss was the only statistically significant factor associated with late mortality during available follow-up identified in exploratory univariate Cox regression. These findings support further investigation of: (1) systematic preoperative IIA stenosis quantification and IIA inclusion planning; (2) perioperative renal risk optimisation; (3) strategies to minimise aortic cross-clamp duration; and (4) intraoperative blood-loss reduction. A multicentre prospective validation study is warranted to confirm and extend these findings.

## Figures and Tables

**Table 1:** Baseline characteristics and comorbidities for the overall cohort.

Characteristic	Overall Cohort (*n* = 170)
Age, years (mean ± SD)	63.8 ± 9.4
Male sex, *n* (%)	157 (92.4)
Smoking, *n* (%)	117 (68.8)
Ischaemic heart disease, *n* (%)	141 (82.9)
Prior myocardial infarction (PICS), *n* (%)	49 (28.8)
History of CVA/stroke, *n* (%)	16 (9.4)
COPD, *n* (%)	65 (38.2)
Arterial hypertension, *n* (%)	130 (76.5)
Chronic kidney disease, *n* (%)	31 (18.2)
Diabetes mellitus, *n* (%)	17 (10.0)
LVEF, % (mean ± SD)	61.5 ± 9.3
Carotid artery stenosis ≥ 50% (either side), *n* (%)	22 (12.9)

LVEF: left ventricular ejection fraction; IIA: internal iliac artery; PICS: postinfarction cardiosclerosis; COPD: chronic obstructive pulmonary disease; CVA: cerebrovascular accident. Of the 22 patients with carotid artery stenosis ≥ 50%, 17 (77%) had no prior history of stroke (asymptomatic stenosis at the time of the index procedure); the remaining 5 had a prior stroke (see History of CVA/stroke, above). Right- and left-sided IIA stenosis severity are not summarised in this table because overall-cohort medians and IQRs cannot be derived from the previously reported subgroup-level summary statistics. IIA stenosis severity is instead analysed as a continuous predictor in the regression analyses (Section 3.3.2) and is reported by treatment-strategy subgroup in Supplementary Table S2. A descriptive comparison of all other characteristics above by treatment strategy (urgent vs. staged surgery) is also provided in Supplementary Table S2.

**Table 2:** IIA inclusion category and reconstruction type for the overall cohort.

IIA Inclusion Category	Overall Cohort (*n* = 170)
IIA: antegrade inclusion, *n* (%)	86 (50.6)
IIA: retrograde inclusion, *n* (%)	70 (41.2)
IIA: non-inclusion, *n* (%)	14 (8.2)
**Reconstruction type**	***n *** **(%)**
Bifurcation aorto-femoral bypass	83 (48.8)
Linear aorto-femoral bypass	37 (21.8)
Bifurcation aorto-ilio-femoral bypass	10 (5.9)
Bifurcation aorto-femoral bypass with IIA revascularisation	40 (23.5)

IIA: internal iliac artery. Aortic cross-clamp time (51.6 ± 22.4 min) and blood loss (647 ± 564 mL) are reported for the whole cohort (not available separately by IIA inclusion category). Reconstruction type was recorded for all patients and determined the IIA inclusion category (see Section 2.4); proximal anastomotic configuration and the specific IIA revascularisation technique were not recorded separately (see Limitations, Section 4.6). IIA inclusion category was included as a covariate in the mortality analyses. A descriptive comparison of the IIA inclusion-category rows above by treatment strategy (urgent vs. staged surgery), including the χ^2^ test across the three categories (*p* < 0.001), is provided in Supplementary Table S3.

## Data Availability

The data presented in this study are available from the corresponding author upon reasonable request. Data are not publicly available due to ethical restrictions and national patient confidentiality regulations of the Republic of Kazakhstan.

## Supplementary Materials

The following supporting information can be downloaded at: https://www.mdpi.com/article/10.3390/medicina62102001/s1, Figure S1: ROC curves for predictors of 30-day mortality after open aorto-iliac reconstruction; Table S1: Summary of ROC analysis for individual predictors of 30-day mortality; Table S2: Baseline characteristics and comorbidities by treatment strategy (urgent surgery without staged preoperative comorbidity correction, Group 1, n = 86; staged surgery with preoperative comorbidity correction, Group 2, n = 84 ); Table S3: IIA inclusion category by treatment strategy (urgent surgery without staged preoperative comorbidity correction, Group 1, n = 86; staged surgery with preoperative comorbidity correction, Group 2, n = 84).

## Abbreviations

The following abbreviations are used in this manuscript:

**AIOD**	Aorto-iliac occlusive disease
**AIR**	Aorto-iliac reconstruction
**IIA**	Internal iliac artery
**CKD**	Chronic kidney disease
**CAD**	Coronary artery disease
**LVEF**	Left ventricular ejection fraction
**OR**	Odds ratio
**HR**	Hazard ratio
**CI**	Confidence interval
**PICS**	Postinfarction cardiosclerosis
**COPD**	Chronic obstructive pulmonary disease
**CABG**	Coronary artery bypass grafting
**PTCA**	Percutaneous transluminal coronary angioplasty
**MSCTA**	Multi-slice computed tomography angiography
**TASC II**	Trans-Atlantic Inter-Society Consensus II
**STROBE**	Strengthening the Reporting of Observational Studies in Epidemiology
