# Supplementary material for: Predictors of Early Mortality After Open Aorto-Iliac Reconstruction in Patients with Aortoiliac Occlusive Disease: An Observational Cohort Study
Source: Medicina (Kaunas). 2026 Oct 16;62(10):2001. doi: 10.3390/medicina62102001 (PMC13707333; doi:10.3390/medicina62102001)
Supplement: Supplementary file 1 [file medicina-62-02001-s001.zip › medicina-4526975-supplementary.pdf]

Supplementary Materials

Figure S1. ROC curves for predictors of 30-day mortality after open aorto-iliac reconstruction. Dots mark optimal operating points. The categorical analyses used clinically selected thresholds (IIA ≥70%; cross-clamp time ≥60 min), which differed from the ROC-derived optimal cut-offs.

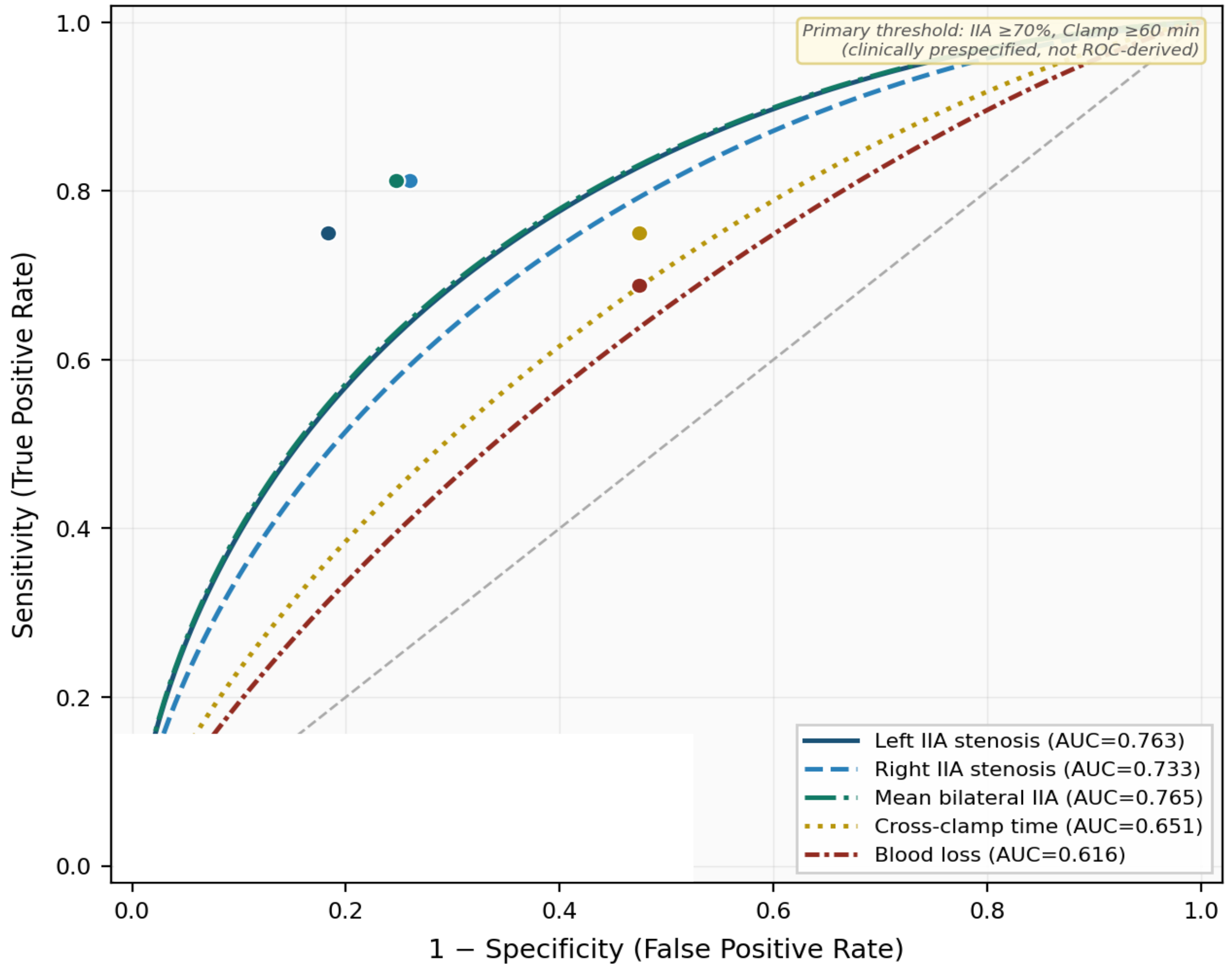

Table S1. Summary of ROC analysis for individual predictors of 30-day mortality (data-derived optimal cut-offs; presented separately from Figure S1 for legibility).

Predictor	AUC	Cut-off	Sensitivity (%)	Specificity (%)
Left IIA stenosis	0.763	65%	75.0	81.7
Right IIA stenosis	0.733	40%	81.2	74.0
Mean bilateral IIA	0.765	50%	81.2	75.3
Cross-clamp time	0.651	50 min	75.0	52.6
Blood loss	0.616	600 mL	68.8	52.6

Table S2. Baseline characteristics and comorbidities by treatment strategy (urgent surgery without staged preoperative comorbidity correction, Group 1, n = 86; staged surgery with preoperative comorbidity correction, Group 2, n = 84). Presented as a descriptive comparison; given the marked baseline imbalance between groups, these between-group differences should not be interpreted causally (see main text, Section 4.6). The overall-cohort baseline characteristics are reported in Table 1 of the main text.

Characteristic	Group 1 (n = 86)	Group 2 (n = 84)	p
Age, years (mean ± SD)	62.6 ± 9.6	65.1 ± 9.1	NS
Male sex, n (%)	77 (89.5)	80 (95.2)	NS
Smoking, n (%)	58 (67.4)	59 (70.2)	NS
Ischaemic heart disease, n (%)	77 (89.5)	64 (76.2)	0.023
Prior myocardial infarction (PICS), n (%)	13 (15.1)	36 (42.9)	<0.001
History of CVA/stroke, n (%)	6 (7.0)	10 (11.9)	NS
COPD, n (%)	37 (43.0)	28 (33.3)	NS
Arterial hypertension, n (%)	54 (62.8)	76 (90.5)	<0.001
Chronic kidney disease, n (%)	3 (3.5)	28 (33.3)	<0.001
Diabetes mellitus, n (%)	7 (8.1)	10 (12.3)	NS
LVEF, % (mean ± SD)	61.8 ± 7.8	61.2 ± 9.5	NS
IIA stenosis right, % (median, IQR)	20 (0–50)	20 (0–50)	NS
IIA stenosis left, % (median, IQR)	25 (0–60)	20 (0–60)	NS
Carotid artery stenosis ≥50% (either side), n (%)	8 (9.3)	14 (16.7)	NS

LVEF: left ventricular ejection fraction; IIA: internal iliac artery; PICS: postinfarction cardiosclerosis; COPD: chronic obstructive pulmonary disease; CVA: cerebrovascular accident; NS: not significant (p ≥ 0.05).

Table S3. IIA inclusion category by treatment strategy (urgent surgery without staged preoperative comorbidity correction, Group 1, n = 86; staged surgery with preoperative comorbidity correction, Group 2, n = 84). Presented as a descriptive comparison; treatment strategy differed significantly between groups for IIA inclusion category (χ^2 test across the three categories, $p < 0.001$). IIA inclusion category was assigned according to the type of aorto-iliac reconstruction (see main text, Section 2.4). Reconstruction type and the overall-cohort figures are reported in Table 2 of the main text.

IIA Inclusion Category	Group 1 (n = 86)	Group 2 (n = 84)
IIA: antegrade inclusion, n (%)	29 (33.7)	57 (67.9)
IIA: retrograde inclusion, n (%)	46 (53.5)	24 (28.6)
IIA: non-inclusion, n (%)	11 (12.8)	3 (3.6)

IIA: internal iliac artery. Aortic cross-clamp time (51.6 ± 22.4 min) and blood loss (647 ± 564 mL) reported for the whole cohort (not separately available by group).

IIA: internal iliac artery; AUC: area under the receiver operating characteristic curve. Cut-off values shown are ROC-derived (data-driven optima specific to this cohort); the categorical multivariable analysis used clinically selected thresholds (IIA ≥70%; cross-clamp time ≥60 min), which differed from these ROC-optimal values.
